# Metabolomics and WGCNA Analyses Reveal the Underlying Mechanisms of Resistance to *Botrytis cinerea* in Hazelnut

**DOI:** 10.3390/genes16010002

**Published:** 2024-12-24

**Authors:** Jun Sun, Liyuan Lu, Juanjuan Liu, Yanhong Cui, Hanqi Liu, Yue Zhang, Zeyang Zheng, Weicong Yang

**Affiliations:** Liaoning Institute of Economic Forestry, Dalian 116031, China; liyuan_lu@163.com (L.L.); juanjuan_9215@163.com (J.L.); cuiyh0722@163.com (Y.C.); 15840167311@163.com (H.L.); jjlzy1982@163.com (Y.Z.); zeyangzheng613@126.com (Z.Z.); 19948657165@163.com (W.Y.)

**Keywords:** hazelnut, *B. cinerea*, metabolomics, transcription factors, WGCNA

## Abstract

Background: Hazelnut (*Corylus*), a significant woody oil tree species in economic forests, faces production constraints due to biotic stresses, with Hazelnut Husk Brown Rot, caused by the pathogenic necrotrophic fungus *Botrytis cinerea* (*B. cinerea*), being the most severe. To date, limited information is available regarding the resistance of hazelnuts to *B. cinerea*. To better understand the mechanisms of resistance to *B. cinerea*. in hazelnut, we conducted metabolomics and WGCNA analyses of a *B. cinerea*-resistant Ping’ou hybrid hazelnut variety (Dawei; DW) and a susceptible variety (Qiuxiang; QX). Methods: In this study, metabolomics and weighted gene co-expression network analysis (WGCNA, weighted correlation network analysis) were applied to elucidate the resistance mechanisms underlying different hazelnut varieties to *B. cinerea*. Our study focused on the metabolome profiles of DW and QX plants after 72 h of *B. cinerea* infection. Results: Venn analysis of QX_0 vs. DW_0 and QX_72 vs. DW_72 revealed 120 differential accumulation metabolites (DAMs) that were upregulated. Among these metabolites, the concentrations of flavonoids and phenolic acids in DW were significantly higher than those in QX, respectively, suggesting that the elevated levels of these compounds contribute substantially to the resistance of hazelnut against *B. cinerea*. 3,4-hydroxyphenyllactic acid and phloretin were significantly more abundant in accumulation in DW than in QX after infection by *B. cinerea*. Conclusions: This study provides that the elevated levels of these compounds (flavonoids and phenolic acids) contribute substantially to the resistance of hazelnut against *B. cinerea*. Furthermore, 3,4-hydroxyphenyllactic acid and phloretin were identified as pivotal metabolites in modulating the resistance of hazelnut to *B. cinerea*. Through WGCNA analyses, we identified four transcription factors (WRKY19, HSFC1, ERF071, and RAP2-1) that are most likely to regulate the synthesis of 3,4-dihydroxyphenyllactic acid and phloretin. This study provides crucial insights for further investigation into the regulatory network of metabolites associated with hazelnut resistance to *B. cinerea*.

## 1. Introduction

Hazelnut (*Corylus*), belonging to the *Betulaceae* family, is commonly seen as an understory plant in mixed-forest environments. It is the fifth most significant tree nut worldwide, noted for its large cultivation regions and high yield potential [1]. However, its cultivation is limited by biotic stresses, primarily Hazelnut Husk Brown Rot caused by the pathogenic necrotrophic fungus *B. cinerea* [2]. Commonly referred to as gray mold, *B. cinerea* is a highly detrimental plant pathogen with a broad host range, capable of infecting over 200 crop species worldwide, causing substantial agricultural losses [3,4]. The fungus not only manipulates the plant’s programmed cell death mechanisms to infect vegetative tissues but also impacts flowers and fruit [3,5]. Research has demonstrated that *B. cinerea* can colonize plants systemically without leading to disease symptoms [6]. *B. cinerea* is now recognized as one of the most thoroughly researched necrotrophic plant pathogens [4]. Even though fungicides can be used for management, there is increasing worry about resistance developing to these chemical treatments, which also raises public concerns about food safety [7]. Although investigations have been made into the impact of fungal pathogens on plants, there is a lack of in-depth findings on the molecular defense mechanisms of hazelnuts against *B. cinerea*. Thus, clarifying how hazelnuts defend against this pathogen is essential for enhancing our knowledge of the possible molecular defense strategies of these fruits. The study found that Hazelnut Husk Brown Rot has occurred seriously in northeast China in recent years. It has been identified as a new disease caused by *B. cinerea*. In severe cases, 50–60% of the husks of the whole tree are browned and decayed, which seriously affects the yield. In order to reduce the occurrence of this disease and improve the resistance of the plants, we carried out a combined transcriptome and metabolome analysis. We screened for genes, metabolic pathways, and metabolites associated with hazelnut disease resistance.

Plants have evolved a sophisticated immune system to defend against *B. cinerea*, involving the production of defense hormones and changes in gene expression [8,9,10,11]. Previous studies have indicated that transcription factors (TFs) are essential controllers of gene expression, significantly impacting the intricate molecular defense network and supporting plant immune responses [12]. So far, numerous defense-associated TFs have been discovered in plants, such as MYBs, AP2/ERFs, NACs, and WRKYs [13,14,15,16]. WRKYs are the most extensive group of transcription regulators in land plants, influencing growth, development, and responses to biotic and abiotic stresses [14,17]. AP2/ERFs are essential for plant disease resistance, integrating hormonal signaling pathways that govern this resistance [13,18,19].

Plant resistance to pathogen infection involves the production of secondary metabolites and the defense hormones salicylic acid and ethylene [20,21]. Moreover, secondary metabolites improve plant defenses by neutralizing reactive oxygen species as antioxidants, being toxic to pathogens, activating defense-related genes [22]. The combination of transcriptomic and metabolomic analyses has recently been effective in revealing the mechanisms that contribute to disease resistance and expression changes in key TFs. TFs are closely linked to the regulation of secondary metabolite synthesis in reaction to biotic stresses [13,14,23,24]. There is growing evidence that particular WRKY and AP2/ERF transcription factors are vital in controlling the biosynthesis of important natural products by altering the expression of genes related to metabolite production [13,25]. For example, the expression of phytoalexin biosynthetic genes in maize is positively regulated by ZmWRKY79, which contributes to the plant’s stress response [26]. The WRKY33-mediated pathway for indolic glucosinolate metabolism enhances resistance to *Alternaria brassicicola* in both *Arabidopsis* and *Brassica* species [27]. Furthermore, NbERF-IX-33 is involved in the regulation of phytoalexin production, granting *Nicotiana benthamiana* resistance against *Phytophthora infestans* [23]. In *Vitis quinquangularis*, VqERF114 regulates stilbene synthesis by engaging with VqMYB35 [21]. A variety of other TFs are responsible for the regulation of secondary metabolites. For instance, TaNAC032 modulates the expression of lignin-biosynthetic genes to enhance resistance against Fusarium head blight in wheat [28]. A study on the R2R3-MYB family in *Arabidopsis* found that MYB34, MYB51, and MYB122 are involved in the production of indolic glucosinolates, whereas MYB28, MYB29, and MYB76 control the production of aliphatic glucosinolates [29,30].

There have been significant attempts to engineer crop plants that can withstand *B. cinerea*, with an emphasis on being environmentally sustainable, safe, and affordable. The hybrid hazelnuts are produced through crossbreeding, with *C. heterophylla* Fisch. serving as the female parent and pollen from various European hazelnut (*C. avellana* L.) seedlings as the male parent [31]. This research focused on two Ping’ou hybrid hazelnut types, DW and QX. Earlier studies showed that DW is resistant to *B. cinerea*, while QX is susceptible to the pathogen [32]. In order to find key metabolites tied to disease resistance, metabolomic analyses were performed on these two hazelnut varieties inoculated with *B. cinerea*. Furthermore, WGCNA was used to shed light on the transcription factor regulatory network managing metabolites related to disease resistance. This study delivers important insights into how hazelnuts defend themselves against *B. cinerea*, thus possibly promoting the genetic improvement of hazelnut varieties with greater resistance to *B. cinerea*.

## 2. Materials and Methods

### 2.1. Plant Materials, Growth Conditions, and B. cinerea Inoculation

The varieties examined include DW, recognized for its disease resistance, and QX, noted for its susceptibility [32]. We selected these two highly resistant and highly susceptible varieties according to their symptoms, incidence rate, and disease index based on the previous disease resistance test. They are Ping’ou hybrid hazelnut varieties (*C. heterophylla* Fisch × *C. avellana* L.) that are 5–6 years old. The experiment was conducted at the Songmudao Base (121°750′ E, 39°400′ N) in Dalian City, Liaoning Province, China, from July 2020 to July 2021. Two varieties of hazelnuts are planted in the same environment. This base is a northern temperate monsoon continental climate, with an average annual air temperature of 9.3 °C, annual precipitation of 623.5 mm, a frost-free period of 169 days, and soil type brown loam. For each variety, 10 fruit bracts were selected and subjected to puncturing. *B. cinerea* Z9 was cultured on potato dextrose agar (PDA) plates at 25 °C for a duration of seven days. Subsequently, a piece of the culture medium containing the bacteria was extracted using a 5 mm hole punch and applied to cover the wounds. Samples were collected at 0, 2, 3, 4, 6, and 8 days post-inoculation (dpi). The samples were sectioned into tissue blocks measuring 1–2 cm and randomly weighed to 2–3 g and stored at −80 °C for further analysis. Each treatment was conducted in triplicate.

### 2.2. Determination of the Physiological and Biochemical Parameters

The activities of superoxide dismutase (SOD) and catalase (CAT) were assessed using the Superoxide Dismutase Activity Assay Kit (BC0175) and the Catalase Activity Assay Kit (BC0205) (Beijing Solarbio Science & Technology Co., Ltd., Beijing, China), respectively. The peroxidase (POD) activity in the plant samples was elevated using the Peroxidase Activity Assay Kit (E-BC-K227-M) (Elabscience Biotechnology Co., Ltd., Wuhan, China). The proline (PRO) content was determined using the Proline Colorimetric Assay Kit (E-BC-K177-M). The enzymatic activity assays, including the establishment and calculation of standard curves, was conducted strictly according to the instructions provided with the kit.

### 2.3. Metabolomic Profile Detection and Analysis

The metabolomic profile was conducted by Norminkoda Biotechnology Co., Ltd. (Wuhan, China) according to standard procedures. The samples were pulverized into a fine powder, and 100 mg of this freeze-dried powder was dissolved in 1.2 mL of a 70% methanol solution. The mixture was vortexed for 30 s at 30 min intervals, repeated six times, and then stored overnight in a refrigerator at 4 °C. For UPLC-MS/MS analysis, the extracts were filtered with a 0.22 μm nylon syringe filter. The sample extracts underwent analysis using an LC-ESI-MS/MS system featuring a UPLC component (Shim-pack UFLC SHIMADZU CBM A system, https://www.shimadzu.com/, accessed on 3 August 2024) and a mass spectrometer (QTRAP^®^ 4500+ System, https://sciex.com/, accessed on 6 August 2024). The identification of metabolites involved using both Metware’s and a public database, while quantitative analysis was performed using Analyst 1.6.3 software. Orthogonal partial least squares discriminant analysis was used to evaluate the different accumulation patterns of metabolites across various samples.

To ensure substantial changes in metabolite abundance are detected, enabling accurate identification of biologically relevant differences while effectively excluding minor fluctuations or noise, we chose a method with a |Log2 Foldchange| threshold of 2 or above for screening DAMs. Metabolites meeting this criterion, along with a VIP score of 1 or above, were labeled as DAMs. Furthermore, pathway enrichment analysis was conducted using TBtools software (v2.142) and the Kyoto Encyclopedia of Genes and Genomes (KEGG) [33].

### 2.4. Hub Gene Identification Using WGCNA

The WGCNA package was employed for weighted correlation network analysis [34]. Transcriptome data (project number PRJNA798644) are derived from previously published articles [35]. For the WGCNA network construction and module detection, 15,947 genes were utilized, with normalization of gene abundances performed beforehand, using default settings. A power β of 9 was selected as the optimal value to improve the similarity matrix, aiding in the creation of a scale-free co-expression network. An evaluation of the correlation between module eigengenes and traits was conducted, defining hub genes as those with a module membership (MM.abs) exceeding 0.8 and a gene significance (GS.abs) more than 0.5. The genes within the most significant module, characterized by a WGCNA edge weight of ≥0.15, were visualized using Cytoscape_version 3.1 [36].

### 2.5. RNA Extraction and RT-qPCR Analysis

Total RNA was extracted from the fruit bracts of hazelnuts, employing the RNAprep Pure Plant Plus kit (TIANGEN Biotech, Beijing, Co., Ltd., Beijing, China). Subsequently, 1 µg of RNA, pre-treated with RNase-free DNase, served as the template for cDNA synthesize. RT-qPCR analysis was conducted using the SYBR Premix Ex Taq (Takara Bio, Inc., Kusatsu, Japan) in conjunction with the CFX96^TM^ Real-Time Detection System (Bio-Rad, Hercules, CA, USA). Specific primers for RT-PCR used in this study were designed utilizing NCBI Primer-BLAST (https://www.ncbi.nlm.nih.gov/tools/primer-blast/ (accessed on 12 November 2024)) (Table S1). Targeted gene expression levels were standardized to *Cha-Actin* [37] and quantified using the 2^−ΔΔCt^ method [38].

### 2.6. Statistics Analysis

Data processing and correlation analysis were both conducted using Excel 2016 and SPSS 19.0 software. Variance analysis and significance testing were performed using the least significant difference (LSD) method, Duncan’s new multiple range test (DNMRT), and *t*-test within SPSS 19.0.

The column stacking diagram was generated utilizing OmicShare Tools (https://www.omicshare.com/tools/, accessed on 15 October 2024) [39]. Heatmaps and clustering analyses were executed using TBtools [40]. Principal component analysis (PCA) was performed in RStudio (version 2023.06.1–524) employing the FactoMineR and factoextra packages [41,42]. Both the column diagram and line chart were produced with GraphPad Prism 5. Venn diagrams were analyzed using SRplot (https://www.bioinformatics.com.cn, accessed on 21 October 2024) [43]. The results for the polar column charts were generated using the CNSknowall platform (https://cnsknowall.com, accessed on 28 October 2024).

## 3. Results

### 3.1. Phenotypic Characteristics and Physiological and Biochemical Parameters of QX and DW Following Inoculation with B. cinerea

**Figure 1:**
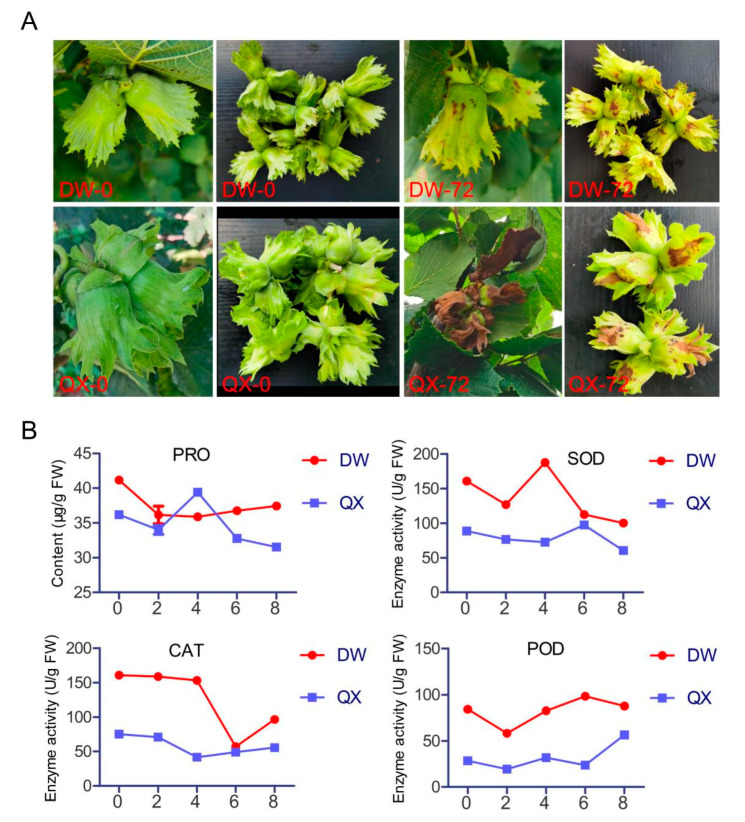
Phenotypic characteristics (**A**) and physiological and biochemical parameters (**B**) of QX and DW following inoculation with *B. cinerea*. The time points 0 and 72 correspond to 0 and 72 hpi, respectively (**A**). The time points 0, 2, 4, 6, and 8 correspond to 0, 2, 4, 6, and 8 dpi, respectively (**B**).

To determine the varying disease resistance between DW and QX varieties, *B. cinerea* infection experiments were conducted. The phenotypic characteristics of the DW and QX varieties were evaluated at 0 and 72 h post-inoculation (hpi), demonstrating that the symptoms in DW, upon inoculation with *B. cinerea*, were less severe than those observed in QX (Figure 1A). Subsequently, four defense-related physiological and biochemical parameters were assessed. The results indicated that the PRO content in DW consistently exceeded that in QX at all time points, except at 4 dpi, and the enzymatic activities of SOD, CAT, and POD were consistently higher in the DW variety compared to the QX variety at all assessed time points, except for CAT activity at 6 dpi, which might account for the disease resistance observed in DW varieties.

### 3.2. An Overview of Metabolomics

**Figure 2:**
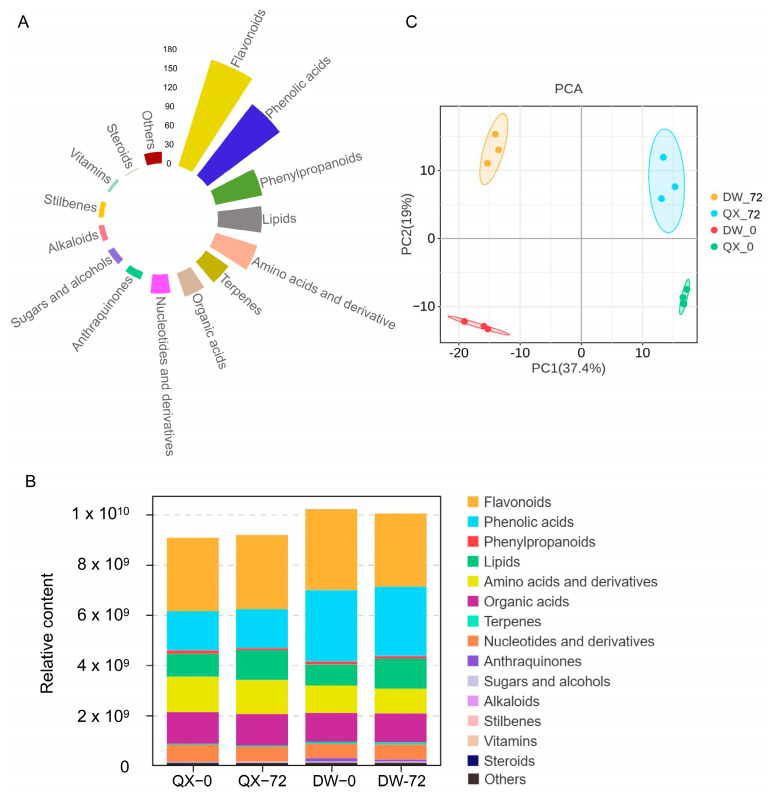
Metabolic profile of QX and DW inoculated with *B. cinerea*. (**A**) Classification of the 705 metabolites detected across four groups (QX_0, QX_72, DW_0, and DW_72). (**B**) Metabolite content across fifteen categories. (**C**) Principal component analysis of metabolite composition across different groups. Each point represents a unique sample, and different colors represent different groups.

To investigate the changes in metabolite content and composition during *B. cinerea* infection, a widely targeted metabolite method involving comprehensive targeted metabolomics approach was performed on the four samples (QX_0, QX_72, DW_0, and DW_72). Following the extraction and deduplication of mass spectral signals, a total of 705 metabolites were identified in both positive and negative ionization modes across the four samples (Table S2). These metabolites were categorized into fifteen distinct classes based on their chemical classification properties, with flavonoids and phenolic acids emerging as the two most prevalent categories (Figure 2A, Table S2). Notably, flavonoids and phenolic acids were also the most abundant compounds within each of the four groups (Figure 2B). Furthermore, the overall metabolite content in DW was higher compared to that in QX at 0 and 72 hpi. Specifically, the phenolic acids content in DW was significantly higher than in QX at both 0 and 72 hpi (Figure 2B, Table S3), suggesting a potential role of phenolic acids in disease resistance. The validity of these findings was corroborated by a principal component analysis (PCA), which revealed that principal component 1 (PC1) and principal component 2 (PC2) accounted for 19% and 37.4% of the variance, respectively (Figure 2C). This analysis effectively distinguished the four samples based on time and variety (Figure 2C). Furthermore, the heat map derived from the hierarchical cluster analysis (HCA) clearly demonstrated that these secondary metabolites could be classified into four distinct subgroups (Figure S1). Consistent with the PCA results, the data indicated significant variations in the secondary metabolite profiles among the groups.

### 3.3. An Overview of DAMs

**Figure 3:**
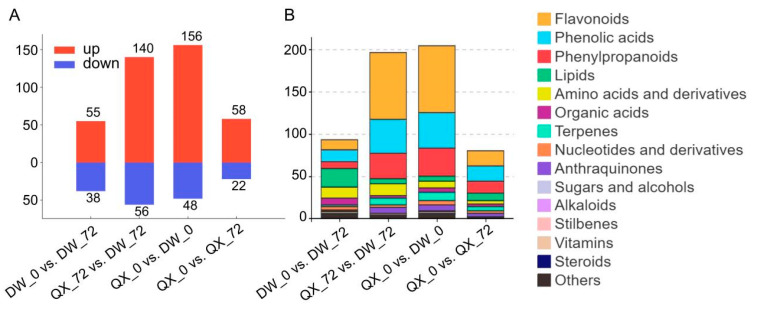
Differential landscape of metabolites in four pairwise comparisons (QX_0 vs. QX_72, DW_0 vs. DW_72, QX_0 vs. DW_0, and QX_72 vs. DW_72). (**A**) Graph illustrates the number of DAMs that are either upregulated or downregulated within these comparison groups. Upregulated DAMs are indicated in red, and downregulated DAMs are shown in blue. (**B**) The numbers of each category within the four groups were derived from a total of 317 DAMs. The control group for each comparison is positioned on the left side.

To enhance the understanding of metabolite accumulation patterns across the four groups, DAMs were identified through four pairwise comparisons: QX_0 vs. QX_72, DW_0 vs. DW_72, QX_0 vs. DW_0, and QX_72 vs. DW_72. The analyses revealed a total of 80 DAMs (22 downregulated and 58 upregulated) in the QX_0 vs. QX_72 comparison, 94 DAMs (55 downregulated and 38 upregulated) in the DW_0 vs. DW_72 comparison, 204 DAMs (156 downregulated and 48 upregulated) in the QX_0 vs. DW_0 comparison, and 196 DAMs (140 downregulated and 56 upregulated) in the QX_72 vs. DW_72 comparison, respectively (Figure 3A, Table S4). In summary, a total of 317 metabolites exhibiting differential accumulation were identified after the elimination of duplicates (Figure 3A and Table S4). Among these metabolites, flavonoids demonstrated the highest degree of variability, followed by phenolic acids and phenylpropanoids (Figure 3B). KEGG pathway analysis revealed that the enriched metabolic pathways were predominantly associated with flavonoid biosynthesis, encompassing pathways such as “flavone and flavonol biosynthesis”, “isoflavonoid biosynthesis”, and “flavonoid biosynthesis”, in addition to the biosynthesis of secondary metabolites (Figure S2).

### 3.4. Venn Analysis of DAMs

**Figure 4:**
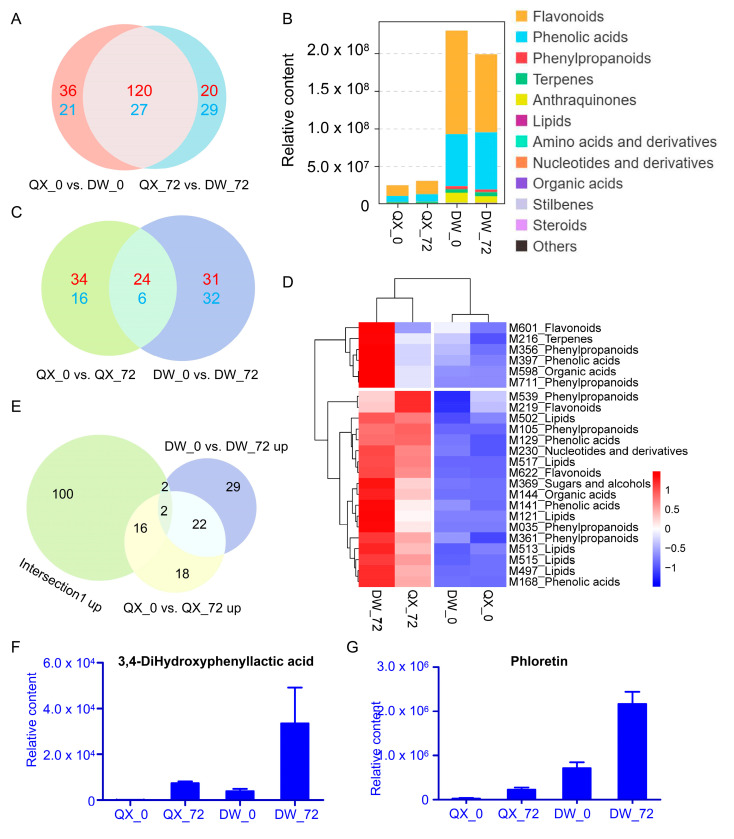
Venn analysis of DAMs. (**A**) Venn analysis of QX_0 vs. DW_0 and QX_72 vs. DW_72. Upregulated DAMs are displayed in red font (defined as intersection 1 up) and downregulated DAMs in blue font. The control group for each comparison is represented on the left side. (**B**) The content of each category derived from 120 upregulated DAMs. (**C**) Venn analysis of QX_0 vs. QX_72 and DW_0 vs. DW_72. Upregulated DAMs are displayed in red font and downregulated DAMs in blue font. The control group for each comparison is represented on the left side. (**D**) Heatmap of 24 upregulated DAMs. (**E**) Venn analysis of intersection 1 up, QX_0 vs. QX_72 up, and DW_0 vs. DW_72 up. The control group for each comparison is represented on the left side. (**F**,**G**) The content of two upregulated DAMs in four groups.

Initially, the differences in metabolites between the two varieties at 0 and 72 hpi were examined. The Venn diagram analysis showed that the intersection of the two comparison groups (QX_0 vs. DW_0 and QX_72 vs. DW_72) contained 147 DAMs (27 were downregulated, and 120 were upregulated) (Figure 4A). Among the 120 upregulated metabolites (categorized as intersection 1 up), flavonoids and phenolic acids emerged as the two most diverse and abundant categories (Figure 4B). Moreover, their content was significantly higher in DW compared to QX at both 0 and 72 hpi (Figure 4B). The findings suggest that the elevated levels of flavonoids and phenolic acids in the DW variety are correlated with its enhanced disease resistance. The Venn diagram analysis of the two comparisons (QX_0 vs. QX_72 and DW_0 vs. DW_72) identified an intersection comprising thirty DAMs (six were downregulated, and twenty-four were upregulated) (Figure 4C). The upregulated DAMs are depicted in a heat map (Figure 4D, Table S4), and these metabolites include flavonoids, phenolic acids, and phenylpropanoids. The Venn diagram analysis revealed that the intersection of intersection 1 up, QX_0 vs. QX_72 up, and DW_0 vs. DW_72 up, contained only two DAMs, namely M397 (3,4-hydroxyphenyllactic acid) and M601 (phloretin), belonging to phenolic acid and flavonoid, respectively. The accumulation of these metabolites was induced by *B. cinerea* infection in both QX and DW varieties, with a more pronounced induction observed in the DW variety compared to QX (Figure 4E–G). This finding suggests a potential association between these metabolites (M397 and M601) and disease resistance.

### 3.5. Gene Screening Using WGCNA

**Figure 5:**
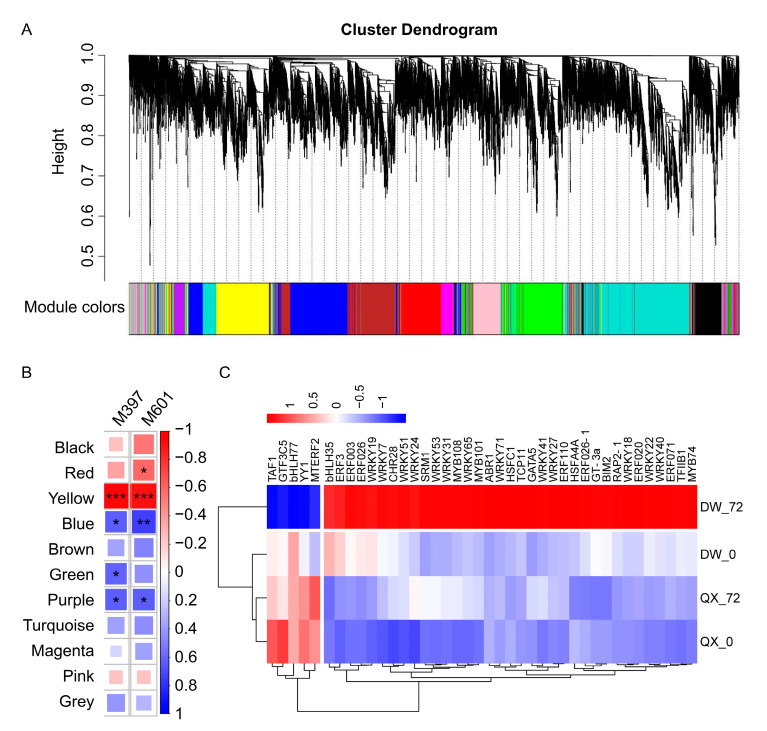
(**A**) Clustering dendrogram of genes. Each leaf corresponds to a gene. A total of 11 merged modules (based on a threshold of 0.20) were identified using a threshold of 0.20 within the framework of a weighted gene co-expression network. (**B**) Heatmap illustrating the correlation between modules and metabolites, with the Pearson correlation coefficient (r) for each module–metabolite pair indicated by color intensity and square size. Asterisks (*, **, ***) denote *p*-values of less than 0.05, 0.01, and 0.001, respectively. Blue hues represent negative correlations, whereas red hues denote positive correlations. (**C**) Heatmap of 40 transcription factor genes within transcriptome profiles.

Utilizing transcriptomic and metabolomic data, a weighted correlation network was constructed, incorporating 15,947 transcripts and two metabolites (M397 and M601), for identifying co-expression modules and hub genes. A soft thresholding power of 20 was selected as it represented the minimum power that appropriately conformed to the scale-free topological index (Figure S3). Following the merged dynamic analysis, a total of 11 modules were identified. These modules included black, red, yellow, blue, brown, green, purple, turquoise, magenta, pink, and grey, containing 939, 1143, 1845, 2081, 1499, 369, 3486, 435, 788, and 1053 genes, respectively (see Figure 5A and Table S5). Notably, the yellow module demonstrated GS values exceeding 0.9 across the two compounds (Figure 5B, Table S6), indicating a marked correlation between the genes in the yellow module and the two metabolites. A total of 966 hub genes were identified within the yellow module (Table S7), including 40 transcription factor genes (Figure 5C, Table S8).

### 3.6. Network and RT-qPCR Analysis of Hub Genes

**Figure 6:**
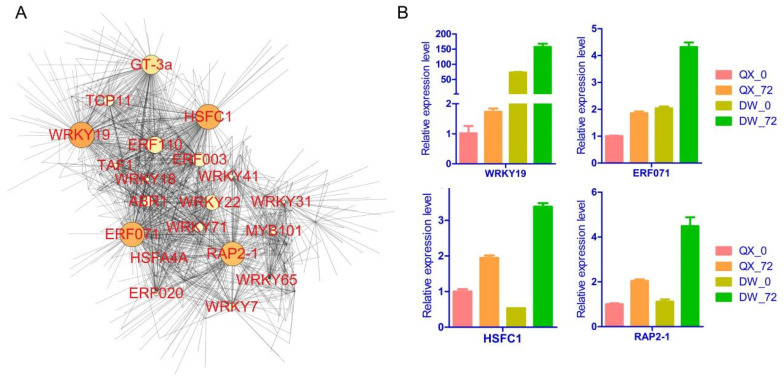
Network and RT-qPCR analysis of hub genes. (**A**) Cytoscape visualization of hub genes within the yellow module. The size of the node represents the number of connected genes. The transparency of the edges reflects the weight value between two genes. (**B**) RT-qPCR analysis of four transcription factor genes (WRKY19, HSFC1, ERF071, RAP2-1).

Utilizing the hub transcription factor genes identified through WGCNA and their associated correlation network, we constructed and visualized a network with a weight threshold greater than 0.15 (Table S9). Through this network analysis, a total of 20 hub transcription factors were identified within the yellow module, with a significant proportion (40%) belonging to the WRKY transcription factor family (Figure 6A). Notably, the findings suggest that four transcription factors (WRKY19, HSFC1, ERF071, and RAP2-1) may play a pivotal role in the biosynthesis of 3,4-hydroxyphenyllactic acid and phloretin. Furthermore, their expression levels were assessed by qRT-PCR analysis to validate the transcriptome profiling results (Figure 6B, Table S10). The findings revealed a high degree of concordance between the RNA-seq and RT-qPCR data, evidenced by a correlation coefficient of 0.977 (Figure S4). The expression levels of the four transcription factor genes were upregulated in response to *B. cinerea* infection in both QX and DW varieties, with a more pronounced induction observed in the DW variety. These observations suggest that the four transcription factors may contribute to the biosynthesis of 3,4-hydroxyphenyllactic acid and phloretin, thereby enhancing the resistance of DW to *B. cinerea*.

## 4. Discussion

Hazelnuts are one of the most commonly grown tree nuts worldwide, known for their large cultivation areas and significant production [1]. Similar to other plants, hazelnuts face numerous biotic stresses throughout their lifecycle [44,45]. *B. cinerea* is known as a highly damaging pathogen among various biotic stresses, causing major decreases in both the yield and quality of hazelnuts [2,4]. *B. cinerea* is capable of infecting over 200 crop species globally, resulting in substantial economic losses in key agricultural sectors [3,4]. There is a shortage of studies on how hazelnuts resist *B. cinerea*, despite extensive documentation of these mechanisms in other plants. Secondary metabolites play a vital role in providing either local or widespread resistance, thus protecting plants from various pathogenic dangers [22]. Therefore, this study explores the alterations in metabolites triggered by *B. cinerea* infection in hazelnuts to discover key metabolites related to disease resistance.

As elucidated in our previous study [35], the results show that the disease symptoms of DW were less severe compared to those of QX at 72 hpi (Figure 1A), which indicated that DW exhibits greater resistance to *B. cinerea* than QX. Previous researches have established that PRO, along with the SOD, POD, and CAT constitute key components of the antioxidant defense system [46,47]. Our results indicate that the level of PRO and the activities of SOD, CAT, and POD in DW were consistently elevated relative to those in QX at most time points. This differential expression may underlie the enhanced disease resistance observed in DW varieties.

Among the phytochemicals present in hazelnuts, phenolic compounds constitute the primary specialized metabolites [48]. These compounds have been demonstrated to possess significant biological activities, including antioxidant, anti-proliferative, and antimicrobial effects [49,50,51]. To examine the variations in metabolite content and composition during infection by *B. cinerea*, a widely targeted metabolomics approach was employed. The results of the metabolomics analyses indicated that flavonoids and phenolic acids are the two most abundant compounds across the four groups analyzed, respectively (Figure 2B). Previous studies indicated that phenolic acids play a crucial role in inducing resistance in plants. For example, cinnamic acid, ferulic acid, and gallic acid were induced by Rhizobia in rice and demonstrated resistance to *Rhizoctonia* [52]. In this study, the phenolic acid content in the DW variety was much higher than that in the QX variety before and after inoculation with *B. cinerea* (Figure 2B, Table S3), suggesting a potential role of phenolic acids in disease resistance.

In response to microbial attacks, plants activate defense mechanisms that result in the induction of a wide range of antimicrobial compounds, some of which may be specific to certain species [22]. Flavonoids constitute a significant class of secondary metabolites with diverse functions in plants, and their crucial roles in pathogen defense have garnered increasing scholarly attention [53]. The immune inducer ZNC enhances tomato resistance to *B. cinerea* by promoting flavonoid synthesis [54]. A new study demonstrated a novel disease defense mechanism by which the WRKY–MAPK pathway promotes flavonoid biosynthesis to defend against pathogen infection [55]. For instance, the concentrations of naringenin and sakuranetin, which function as phytoalexins, significantly increased in rice leaves in response to *Pyricularia oryzae* infection [56]. DAM analysis across four pairwise comparisons (QX_0 vs. QX_72, DW_0 vs. DW_72, QX_0 vs. DW_0, and QX_72 vs. DW_72) revealed that flavonoids exhibited the greatest variability, followed by phenolic acids and phenylpropanoids (Figure 3B). Furthermore, a total of 120 metabolites were found to be upregulated in two comparisons: QX_0 vs. DW_0 and QX_72 vs. DW_72. Notably, the concentrations of flavonoids and phenolic acids were significantly higher in the DW variety compared to the QX variety at 0 and 72 hpi (Figure 4B). These findings suggest that the elevated levels of flavonoids and phenolic acids in the DW variety may contribute to its enhanced disease resistance.

A total of 24 metabolites were upregulated in both the QX_0 vs. QX_72 and DW_0 vs. DW_72 comparisons, indicating that these metabolites are likely induced by *B. cinerea* infection in both the DW and QX varieties. Furthermore, among the 24 identified metabolites, the concentrations of 3,4-dihydroxyphenyllactic acid, also known as homoprotocatechuic acid and phloretin, which are classified as a phenolic acid and a flavonoid, respectively, were significantly elevated in DW compared to QX (Figure 4E–G). These findings imply that 3,4-dihydroxyphenyllactic acid and phloretin may play a crucial role in enhancing the resistance of DW to *B*. *cinerea*. Previous research has demonstrated that apple lines overexpressing MdUGT88F1, an enzyme that catalyzes the glycosylation of phloretin, exhibit increased levels of phlorizin and enhanced resistance to necrotrophic pathogens [57]. Transcriptomics and metabolomics analyses have demonstrated the induction of phloretin biosynthesis in response to infection by *Colletotrichum gloeosporioides*. Furthermore, in vitro assays have indicated that phloretin effectively inhibits the mycelial growth of *C. gloeosporioides* [58].

WGCNA, a widely utilized systems biology approach for analyzing high-dimensional data such as transcriptomic and metabolomic datasets [34], was employed to identify genes significantly associated with the biosynthesis of various metabolites [59,60,61]. Consequently, WGCNA was conducted to identify the key genes involved in the synthesis of 3,4-dihydroxyphenyllactic acid and phloretin (Figure 5). A total of 966 hub genes were identified within the yellow module (Table S7). Previous studies have demonstrated that TFs are pivotal regulators of gene expression, playing significant roles within the intricate molecular defense network that contributes to plant immunity. Furthermore, TFs have been extensively associated with the regulation of secondary metabolite synthesis in response to biotic stresses [13,14,15,16,23]. Therefore, TFs were identified within these hub genes, and their correlation network was established. Ultimately, 20 transcription factors were identified, with WRKY and AP2/ERF representing the largest proportion (65%). Among these, four transcription factors (WRKY19, HSFC1, ERF071, and RAP2-1) were determined to be the most probable regulators of the synthesis of 3,4-dihydroxyphenyllactic acid and phloretin. Overall, our findings highlight candidate metabolites and their associated transcription factors that may play pivotal roles in the regulatory networks conferring resistance to *B. cinerea* in hazelnut.

## 5. Conclusions

This study elucidates the role of elevated flavonoids and phenolic acids in contributing to the resistance of hazelnuts against *B. cinerea*. Notably, 3,4-hydroxyphenyllactic acid and phloretin emerged as key metabolites in enhancing resistance. Through weighted gene co-expression network analysis (WGCNA), four transcription factors—WRKY19, HSFC1, ERF071, and RAP2-1—were identified as potential regulators of these crucial metabolites. These findings offer significant insights into the metabolic and genetic mechanisms underlying hazelnut resistance, paving the way for the genetic enhancement of hazelnut varieties with improved resistance to *B. cinerea*. We will continue to study the resistance mechanism of hazelnuts to *B. cinerea*. Through gene knockout or gene cloning, the functional verification of the gene will be deeply studied to further improve the disease resistance of hazelnuts.

## Data Availability

The original contributions presented in the study are included in the article/Supplementary Material; further inquiries can be directed to the corresponding author.

## Supplementary Materials

The following supporting information can be downloaded at https://www.mdpi.com/article/10.3390/genes16010002/s1, Figure S1: Heatmap of all metabolites; Figure S2: KEGG analysis of DEGs; Figure S3: Weighted gene co-expression network analysis; Figure S4: The correlation coefficient diagram of RNA-Seq data and RT-qPCR; Table S1: Primers used in this study; Table S2: All metabolites;. Table S3: The content of fifteen categories; Table S4: Differential accumulated metabolite; Table S5: Gene2module; Table S6: Module−trait relationships; Table S7: Hub genes identified by WGCNA; Table S8: 40 transcription factor genes; Table S9: Network analysis of hub genes; Table S10: RT-qPCR.
