# Supplementary figures and images for: Metabolomics and WGCNA Analyses Reveal the Underlying Mechanisms of Resistance to Botrytis cinerea in Hazelnut
Source: Genes (Basel). 2024 Dec 24;16(1):2. doi: 10.3390/genes16010002 (PMC11765503; doi:10.3390/genes16010002)

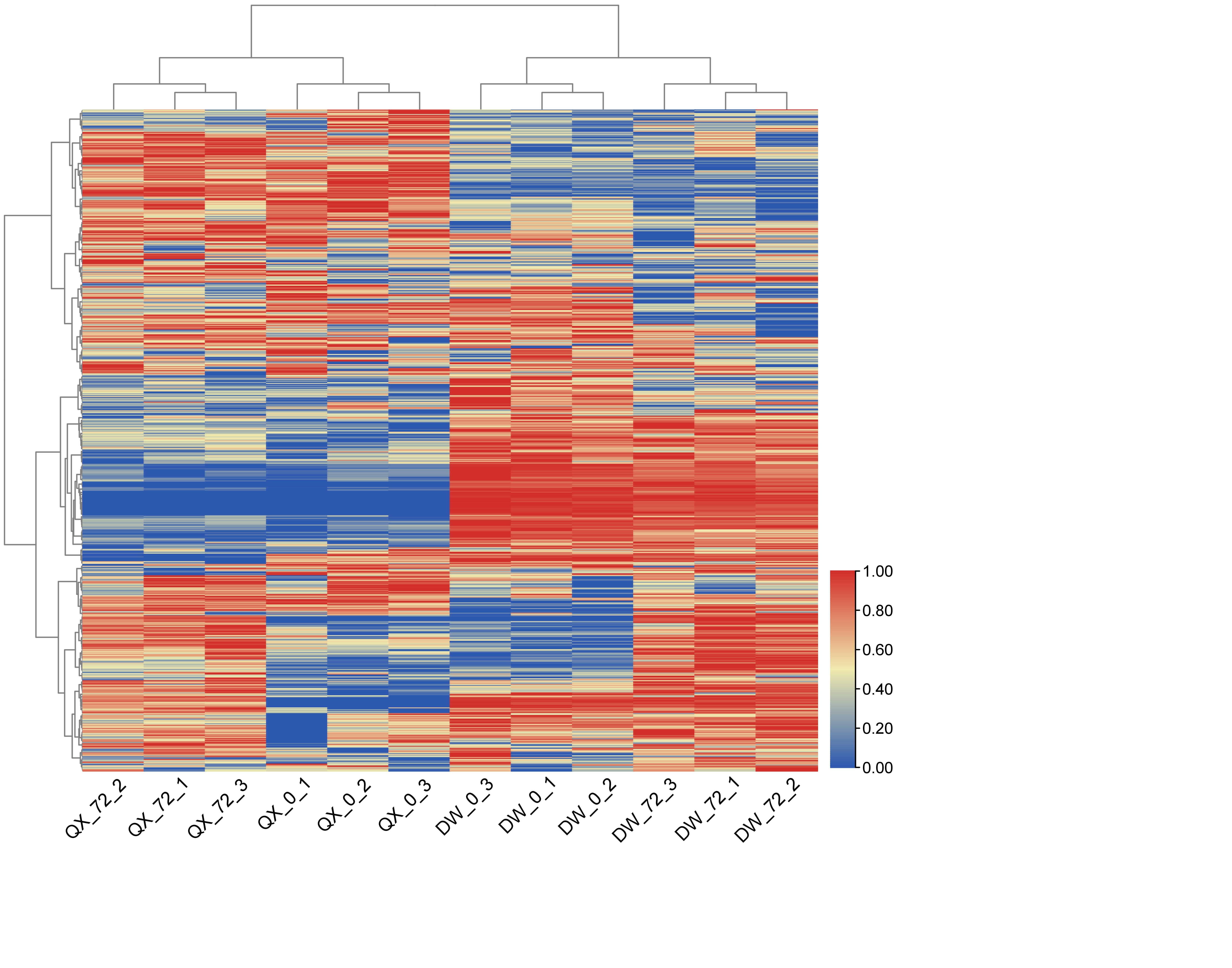

Supplement: Supplementary file 1 [file genes-16-00002-s001.zip › genes-3382460-supplementary/Figure S1.jpg]

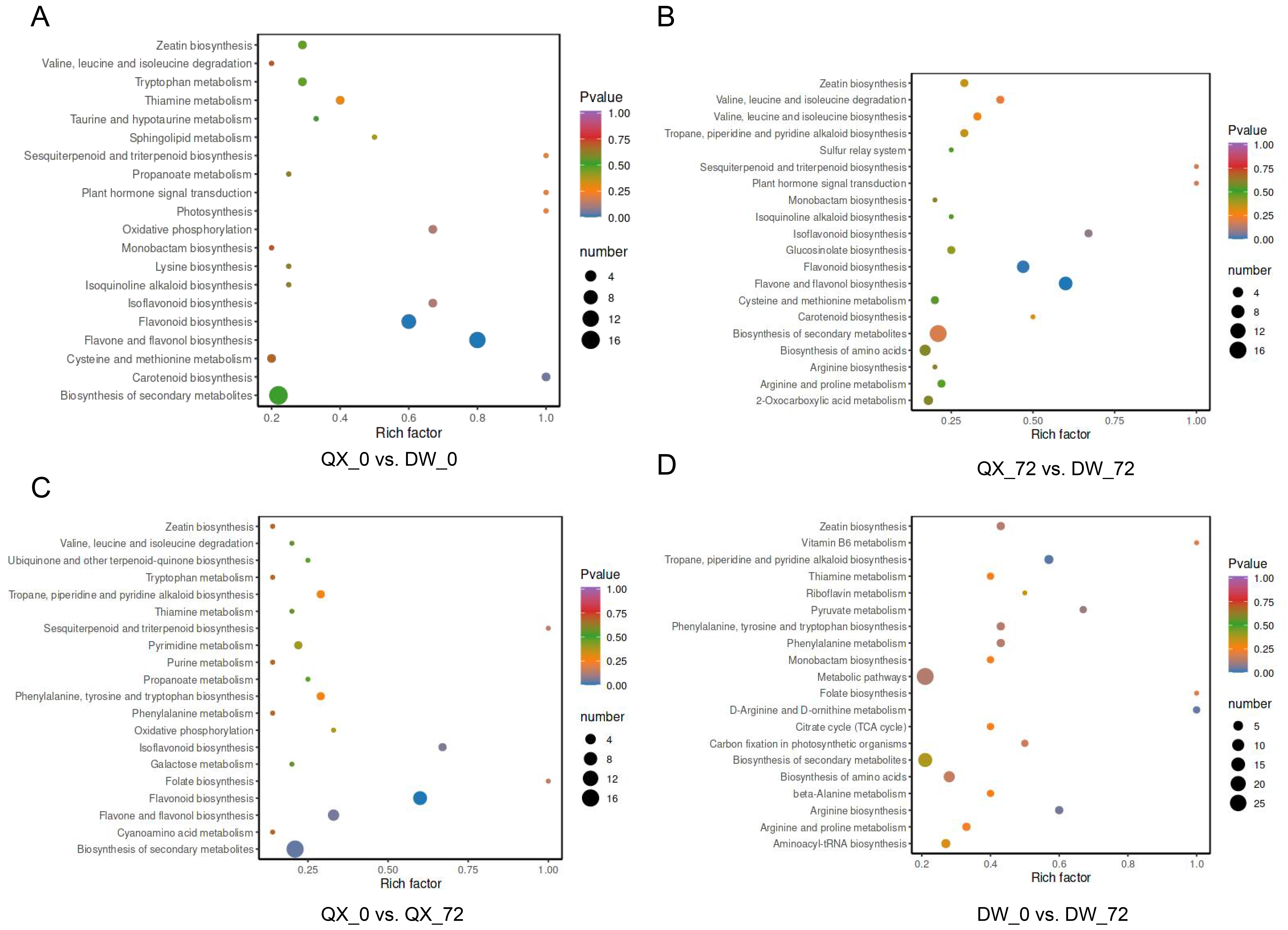

Supplement: Supplementary file 1 [file genes-16-00002-s001.zip › genes-3382460-supplementary/Figure S2.jpg]

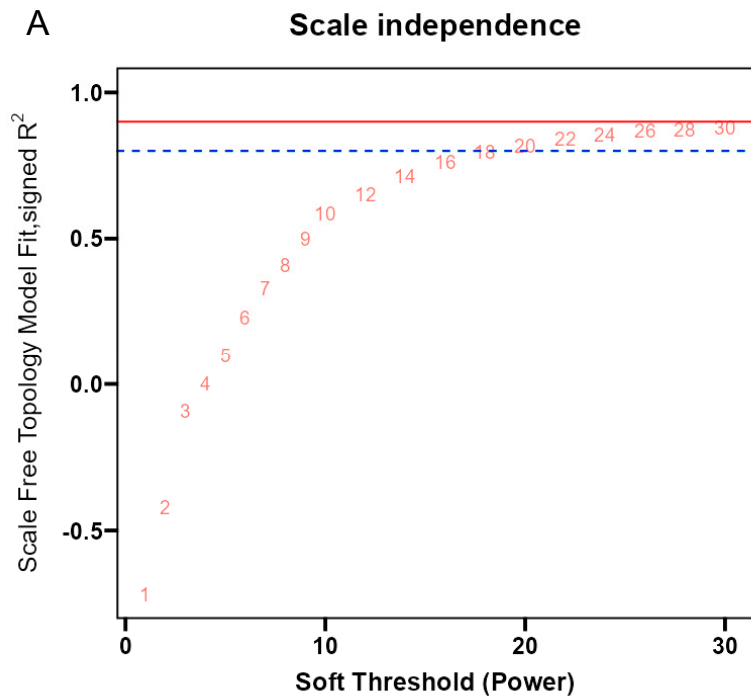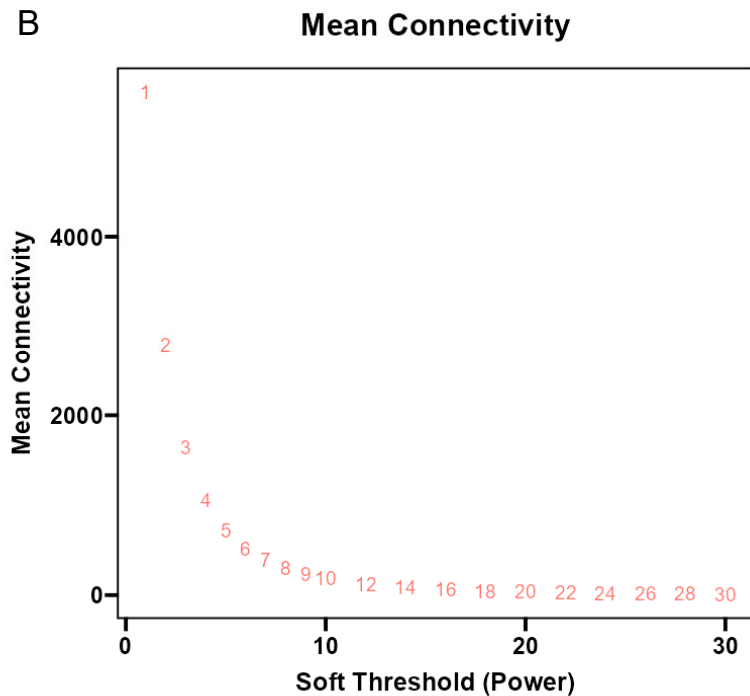

**Figure S3.**

Supplement: Supplementary file 1 [file genes-16-00002-s001.zip › genes-3382460-supplementary/Figure S3.pdf]

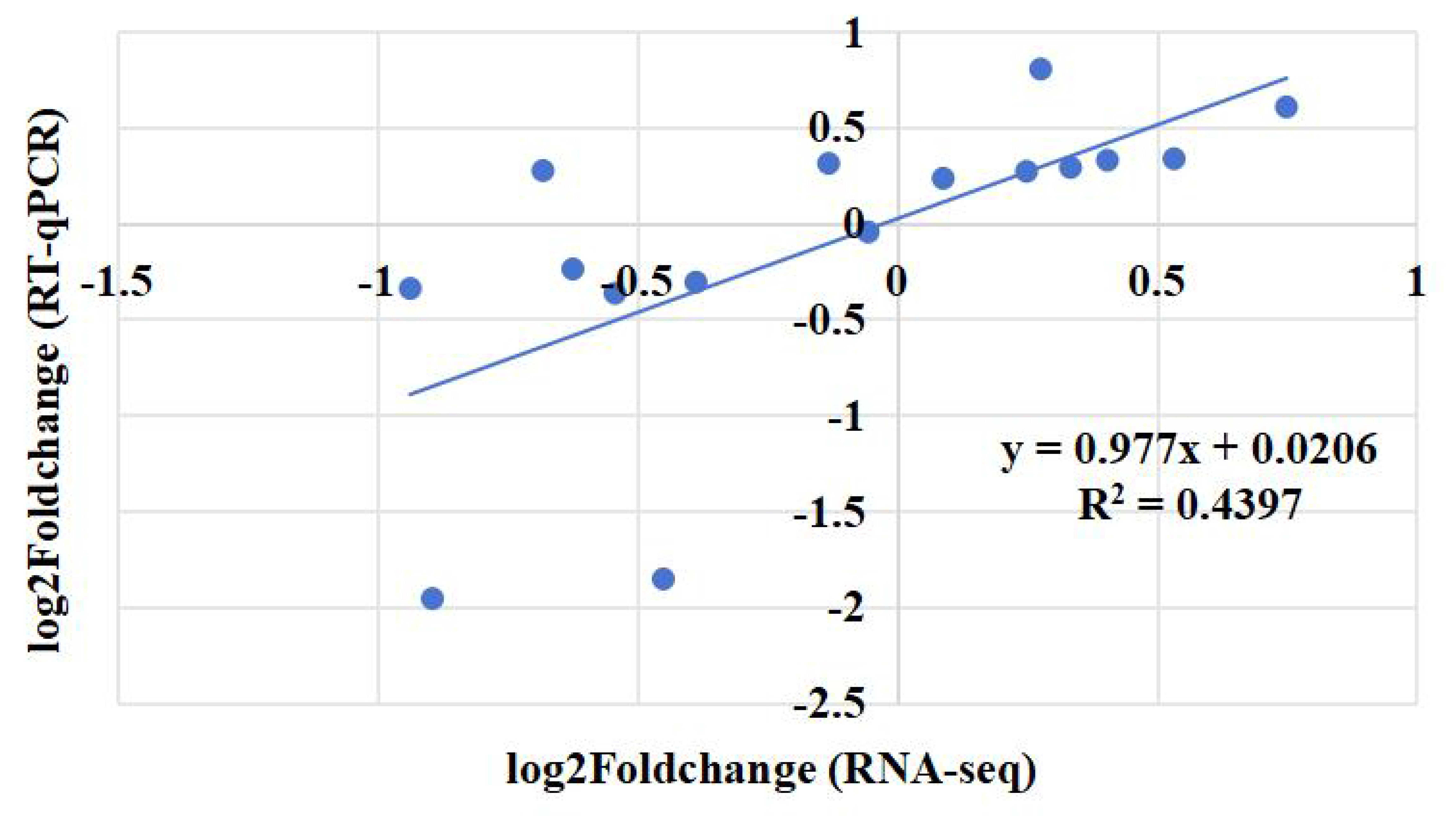

Supplement: Supplementary file 1 [file genes-16-00002-s001.zip › genes-3382460-supplementary/Figure S4.jpg]
